# Semantic Processing Disturbance in Patients with Schizophrenia: A Meta-Analysis of the N400 Component

**DOI:** 10.1371/journal.pone.0025435

**Published:** 2011-10-12

**Authors:** Kui Wang, Eric F. C. Cheung, Qi-yong Gong, Raymond C. K. Chan

**Affiliations:** 1 Neuropsychology and Applied Cognitive Neuroscience Laboratory, Key Laboratory of Mental Health, Institute of Psychology, Chinese Academy of Sciences, Beijing, China; 2 Castle Peak Hospital, Hong Kong Special Administrative Region, China; 3 Huaxi MR Research Centre, Department of Radiology, West China Hospital/West China School of Medicine, Sichuan University, Chengdu, China; Baylor College of Medicine, United States of America

## Abstract

**Background:**

Theoretically semantic processing can be separated into early automatic semantic activation and late contextualization. Semantic processing deficits have been suggested in patients with schizophrenia, however it is not clear which stage of semantic processing is impaired. We attempted to clarify this issue by conducting a meta-analysis of the N400 component.

**Methods:**

Twenty-one studies met the inclusion criteria for the meta-analysis procedure. The Comprehensive Meta-Analysis software package was used to compute pooled effect sizes and homogeneity.

**Results:**

Studies favoring early automatic activation produced a significant effect size of −0.41 for the N400 effect. Studies favoring late contextualization generated a significant effect size of −0.36 for the N400 effect, a significant effect size of −0.52 for N400 for congruent/related target words, and a significant effect size of 0.82 for the N400 peak latency.

**Conclusion:**

These findings suggest the automatic spreading activation process in patients with schizophrenia is very similar for closely related concepts and weakly or remotely related concepts, while late contextualization may be associated with impairments in processing semantically congruent context accompanied by slow processing speed.

## Introduction

### Semantic deficits in patients with schizophrenia

A hallmark of schizophrenia is semantic deficit. The speech of patients with schizophrenia is characterized by loose and aberrant associations, poverty of content and neologisms [1]. Similar phenomena have also been observed in their non-psychotic first-degree relatives [2] and individuals with schizotypal personality features [3]. Semantic deficits have also been considered a potential endophenotype for schizophrenia spectrum disorder [4], [5], [6].

Semantic long-term memory has been posited as an interconnected network, with each node representing a specific concept and the link between nodes representing a certain semantic relationship [7], [8]. Many different theories have been put forward to explain semantic processing in semantic memory. The hybrid three-process theory by Neely and Keefe is believed to be most consistent with experimental data [9]. According to this theory, three different mechanisms explain the processing in semantic memory, namely automatic semantic activation, expectancy and semantic matching. When a node in the semantic network is activated, the activation is not limited to the local site, but also automatically spread to linked nodes. This process is called automatic semantic activation. With the expectancy-based mechanism, a set of lexical candidates is generated in response to a certain semantic context which could be either word or sentence. Semantic matching is a post-lexical process, in which information concerning whether a certain word is semantically related to the previous semantic context is used. Depending on the involvement of attention, semantic processing could be separated into two relatively independent stages, early automatic semantic activation without the involvement of attention and late contextualization (consisting of expectancy and semantic matching) heavily influenced by attention. It has been postulated that the initial spread of activation dominates the first 500 msec of word processing. Late contextualization then comes to play. In this stage, individuals generate reasonable expectancy based on contextual information and integrate old and new information to form a meaningful representation for the whole context. The contextually unrelated materials are inhibited simultaneously. Normal reading includes both early semantic activation and contextualization. In fact, these two stages cannot be totally separated.

A well-established paradigm to investigate semantic processing is the semantic priming task. Behaviorally, the semantic priming effect refers to the reduction of reaction time to a word (e.g., tiger) when it is preceded by a semantically congruent context (e.g. lion) as opposed to a semantically incongruent context (e.g., bread). The semantic context could be either words or sentences. With a relatively short stimulus onset asynchrony (SOA) (i.e., less than 500 msec) in word-pair studies, the priming effect is mainly attributed to early automatic semantic activation. With a long SOA in word-pair studies or in studies using sentence context (also building up over a longer period), the priming effect is mainly attributed to late contextualization processes [9]. It is however important to note that SOA is not the only variable that influences the semantic priming effect. Variables such as relatedness proportion and experimental task are also important [9]. It is believed that a relatively small proportion of related prime-target pairs and experimental tasks which direct participants' attention on other aspects than the semantic relationship between prime and target contribute more to early automatic semantic activation. In contrast, a relatively large proportion of related prime-target pairs and experimental tasks which direct participants' attention to the semantic relationship between prime and target favor late contextualization. In addition, indirect semantic priming effect is also believed to favor early automatic semantic activation (e.g., “lion” primed “stripe” via tiger).

A common way to compare semantic processing between individuals with and without schizophrenia is to compare their semantic priming effect. However, behavioral semantic priming effects are plagued by inconsistencies. As pointed out by a number of qualitative and quantitative reviews, every possible pattern of behavioral semantic priming effect had been reported [10], [11], [12]. The heterogeneous nature of participants might be an important reason for the inconsistent behavioral results [11]. Another important reason could be the non-specific nature of reaction times, which measure the time between the presentation of stimulus and a button press. To delineate the exact nature of semantic deficits in patients with schizophrenia, it is therefore important to target semantic processing directly. Event-related potential (ERP), with its high temporal resolution at the millisecond level, is useful to investigate the nature of semantic deficits in schizophrenia.

### The N400

The behavioral semantic priming effect has a counterpart in event-related potential studies, namely the N400, a negativity peaking at about 400 milliseconds after stimulus onset. Words preceded by a semantically unrelated word or sentence elicit a larger N400 than those followed by related words or sentences [13]. The N400 elicited by words and by sentences are very similar, suggesting a similar underlying mechanism for semantic processing of word and sentence [14]. The N400 has also been used to investigate early automatic semantic activation when the experimental design favors this stage of semantic processing (e.g., with SOA shorter than 500 ms). N400 amplitudes have also been found to be correlated with expectancy of target words in healthy volunteers (r = 0.90, see Kutas, 2011 for a review).

The N400 has been observed to be highly correlated with some features in schizophrenia, such as positive thought disorder (r = 0.41−0.70) [15], [16], [17], [18], [19], [20]. Correlations between measures of N400 and psychotic symptoms (hallucinations and delusions) [21], [22], negative symptoms [16], [21], [23], [24], hallucinatory behavior [25], avolition [26], hostility-suspiciousness [27], withdrawal-retardation [27] and severity of delusion [28] have also been reported. These findings suggest that semantic deficits are related to a variety of symptoms in schizophrenia.

Many studies have reported N400 abnormalities in patients with schizophrenia [29]. Recently, Kuperberg and colleagues (2010) systematically reviewed N400 data at word, sentence, and discourse levels. Based on the two-stage semantic processing theory, the accumulation of ERP data has now enabled us to carry out a meta-analysis between schizophrenia and normal controls to examine the nature of semantic processing deficit in patients with schizophrenia.

### Purpose of the study

The present study aimed to examine the profile of semantic processing in patients with schizophrenia using the N400 measures within a theoretical model of semantic processing. We grouped the results into two main categories. Effect sizes obtained from word-pair studies with a SOA shorter than 500 ms were taken to reflect the early automatic activation. Effect sizes obtained from word-pair studies using a SOA longer than 500 ms, or from studies using sentence context were taken to reflect late contextualization. However, as SOA is not the only experimental variable that influences semantic priming effect, a number of studies using SOA shorter than 500 ms at the word level but were not designed to examine early automatic activation [18], [24] were excluded from the meta-analysis. The corresponding findings were considered in the discussion section. Two additional studies addressing semantic processing using picture matching tasks were included in the meta-analysis procedure [5], [30], since picture matching and word matching are assumed to share the same mechanism in semantic memory. Four effect sizes were computed to examine the pattern of semantic processing impairments in patients with schizophrenia for each of the components: N400 peak latency, N400 effect (the difference in the N400 amplitudes between congruent/related and incongruent/unrelated conditions), N400 amplitudes for congruent/related conditions and N400 amplitudes for incongruent/unrelated conditions.

## Methods

### Literature search

**Figure 1:**
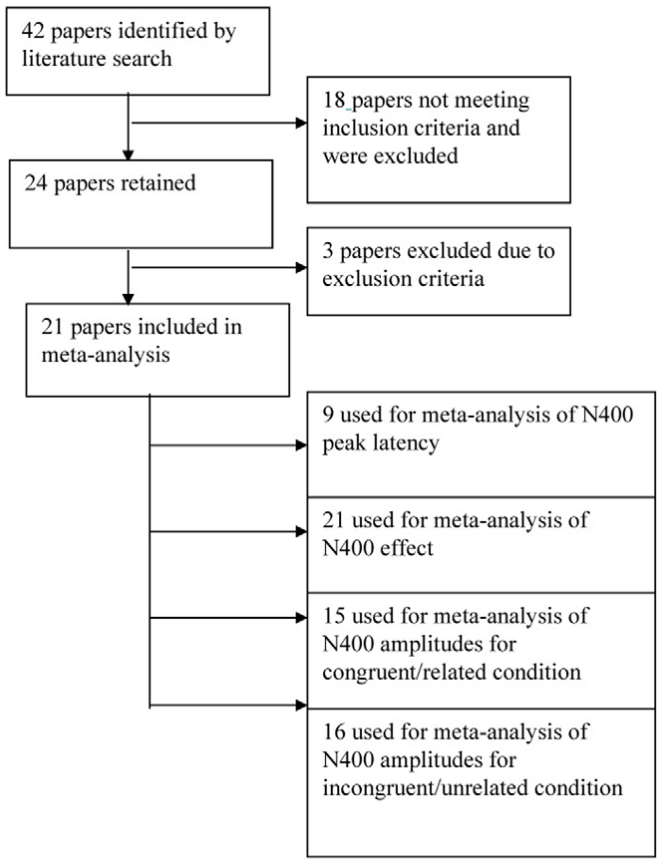
Flowchart for the inclusion of published data for the current meta-analysis.

The flowchart of data extraction for the meta-analysis of each N400 measure is shown in Figure 1. Potential articles were identified through a comprehensive literature search using the databases of EBSCOhost (Academic Search Complete, Medline, and PsychINFO) between January 1980 and October 2010. Two sets of key words were used: “semantic + ERP + schizophrenia” and “semantic + ERP + schizophrenic”. Additional articles were obtained from the reference lists of the initial article base. These search procedures yielded an initial pool of 42 potential articles for inclusion (a complete reference list of all studies is available upon request).

For the meta-analysis, two inclusion criteria were used to select studies in the initial pool for quantitative analysis. These were (1) inclusion of N400 measures in both patients with schizophrenia and normal controls; (2) availability of means and standard deviations or exact t values or F values on at least one of four N400 measures. After this procedure, 24 articles were retained. Thereafter the retained articles were subject to several exclusion criteria, which are listed below:

1. Published in a language other than English [31]
2. The same set of data was reused as documented by the author in a later study [32]
3. Case reports [33]

As a result, 21 articles were retained for the current meta-analysis. Among these, one had two different schizophrenia and control groups [23], [30], and another two had two different experimental conditions in different blocks [22], [34]. For these three papers, each experiment was taken as an independent study for meta-analysis, making a total of 25 valid datasets for meta-analysis of the ERP measures. The patients in all these studies were diagnosed according to different versions of the Diagnostic and Statistical Manual (DSM). (The studies used for the meta-analysis of each N400 measure can be found in the supporting information Table S1).

### Meta-analytical procedure

In general, mean amplitudes were used. When the mean amplitudes were not available, the peak amplitudes were used instead [46]. For studies containing both direct and indirect semantic priming, only data for indirect semantic priming were used. For studies considering the influence of different degree of expectancy or stimulus proportion on semantic priming effect in one single experimental block, only one representative experimental condition was chosen for meta-analysis to avoid over-evaluating a paper. The peak latency in the difference wave was used for computing effect sizes. Some data were obtained by contacting the respective authors directly.

All analyses were performed using the Comprehensive Meta-Analysis (CMA) software package. Effect sizes (Cohen's d) indicating the difference between schizophrenia patients and healthy controls were calculated on the basis of reported statistics (the mean of the schizophrenia sample minus the mean of the healthy control group, divided by the pooled SD). When means and SDs were not available, effect size d was computed from t or F values or estimated from exact P values. Standard meta-analytic methods were adopted to obtain mean effect sizes weighted for study variance and averaged across primary studies [35]. The random model was used for calculating the effect sizes. The stability of the mean effect was estimated by its 95% CI. In addition, the homogeneity statistic, I-squared, was calculated to test whether individual effect sizes for any given variable reflect a single common population effect size.

To address possible ‘file-drawer problem’ [36], a fail-safe number estimating the number of unpublished studies with nil or minimal effect sizes required to reduce an overall effect size to some specified negligible value [36], [37] was calculated. Finally, moderator variables evaluated in relation to both uncorrected and corrected effect sizes including medication status and medication dosage in chlorpromazine equivalent (CPZ) were calculated as well.

## Results

### Peak latency

Nine studies were included in this part of the meta-analysis. The sample size in the schizophrenia group and control group were 149 and 147 respectively. The meta-analysis procedure produced a large effect size of 0.65. This effect was significant as tested by the test of null (Z = 4.32, p<0.001), indicating that the patient group had a larger peak latency compared to controls. The variance was 0.02, with a 95% CI of 0.36–0.95. The fail safe N was 61, which was sufficiently large to make the existence of large numbers of unpublished negligible findings unlikely. The test of heterogeneity generated a non-significant effect, I-squared = 35.18%, p = 0.14, indicating that the studies were homogeneous. The general results are shown in Table 1.

**Table 1:** The general results of the meta-analysis for N400 measures with SOA as moderator.

N400 measures		K	NP	NC	D	Z	P	95% CI	Heterogeneity	
									I-squared	P
N400 Peak latency	All	9	149	147	0.65	4.32	<0.001	0.36–0.95	35.18%	0.13
	Long SOA	6	100	98	0.82	5.51	<0.001	0.53–1.11	0.00%	0.49
N400 effect (difference wave)	All	21	375	365	−0.64	3.97	<0.001	−0.97−−0.31	77.94%	<0.001
	Short SOA	6	137	125	−0.41	3.29	0.001	−0.66−−0.17	4.96%	0.38
	Long SOA	11	177	181	−0.36	3.33	0.001	−0.58−−0.14	4.73%	0.40
N400 Amplitudes for congruent/related conditions	All	15	241	241	−0.55	−2.98	<0.01	−0.92−−0.19	73.47%	0.00
	Short SOA	5	84	84	−0.17	0.97	0.33	−0.52−0.17	50.32%	0.09
	Long SOA	7	105	107	−0.52	−3.35	<0.001	−0.83–0.22	0.00%	0.56
N400 Amplitudes for incongruent/unrelated conditions	All	16	261	261	−0.01	−0.11	0.91	−0.24−0.22	42.64%	0.04
	Short SOA	5	82	82	0.24	1.19	0.23	−0.16−0.64	53.98%	0.07
	Long SOA	10	166	168	−0.15	−1.0	0.30	−0.43−0.13	29.70%	0.17

D = effect size; K = number of studies used for meta-analysis; NC = number of Controls; NP = number of patients; short SOA = less than 500 ms; long SOA = larger than 500 ms at word level or at sentence level; SZ = schizophrenia.

Six studies adopted a long SOA (word pair studies with a SOA longer than 500 ms, or studies using sentences) which included data for 100 patients and 98 normal controls. An effect size of 0.82 was obtained which was significant as indicated by the test of null (Z = 5.51, p<0.001). The variance was 0.02 with a 95% CI of 0.53–1.11. All of the studies were homogenous (I-squared = 0.00%, p = 0.49). Only data from two studies using a SOA shorter than 500 ms were available for meta-analysis of the N400 peak latency. Due to the limited number of studies, no effect size was computed. A total of eight studies were identified using a SOA shorter than 500 ms [16], [18], [22], [24], [25], [34], [38], [39], [40] and all of them used semantic priming at the word level (excluding the study by Mathalon and colleagues which used the picture-word verification task). Some studies conducted an analysis on peak latency, while others did not. Overall, only one study reported a significantly larger peak latency in patients with schizophrenia [38].

### N400 effect (Amplitude of difference wave between congruent/related and incongruent/unrelated condition)

Twenty-one studies were included in this part of the meta-analysis. The sample size in the schizophrenia group and control group were 375 and 365 respectively. Random model meta-analysis produced a medium effect size of −0.64, which was significant as tested by the test of null (Z = 3.97, p<0.001), indicating that patients with schizophrenia had decreased N400 effects compared with controls. The variance was 0.03 and the 95% CI was −0.97−−0.31. The fail safe N was 325. These studies was not homogenous (I-squared = 77.94%, p<0.001).

**Figure 2:**
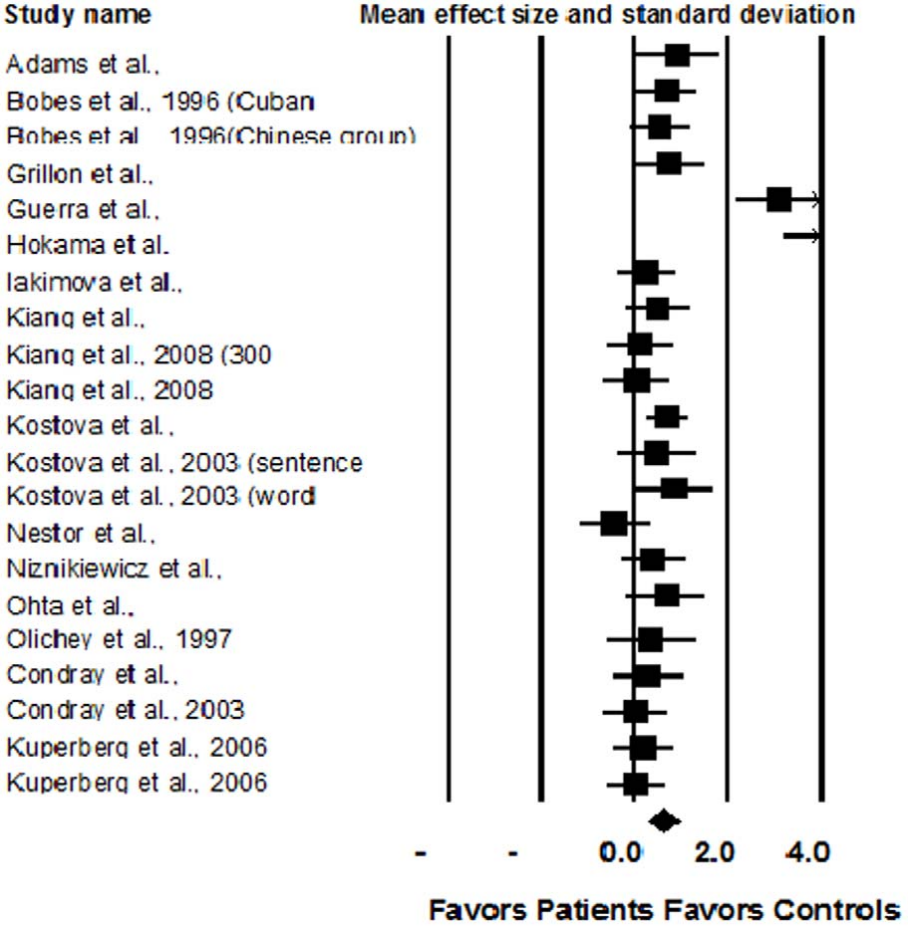
Comparison of the N400 effect between schizophrenia and controls.

**Figure 3:**
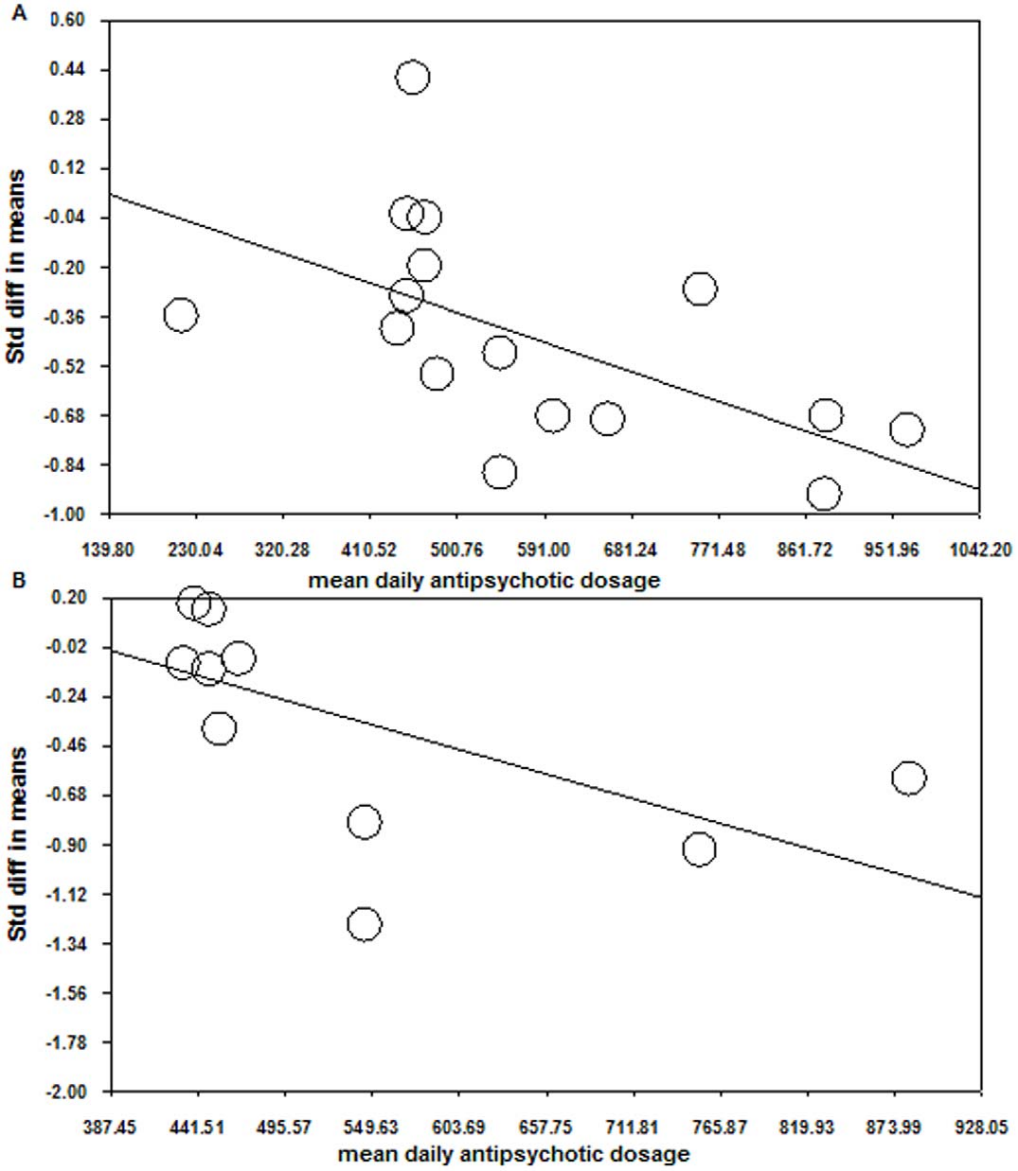
Regression of mean daily antipsychotic dosage on effect sizes of A: N400 effect (upper panel) and B: N400 amplitudes for congruent/related conditions (lower panel).

As shown in Figure 2, studies by Hokama et al. (2003) and Guerra et al. (2009) had extremely large effect sizes. After excluding these two studies, the remaining studies became homogeneous (I-squared = 0.00%, p = 0.57). The effect size was −0.39, significant as tested by the test of null (Z = −4.95, p<0.001). The variance was 0.006, and the 95% CI was −0.55−−0.24. The fail safe N was 97. In the subsequent analysis of different moderators, the studies by Hokama et al. (2003) and Guerra et al. (2009) were excluded. Patients in the study of Hokama et al. (2003) were unmedicated, while Guerra et al. (2009) used pictures as experimental materials and selected only patients with paranoid schizophrenia. These might have accounted for the large effect sizes observed. Six studies had a SOA shorter than 500 ms (d = −0.41) and 11 had a SOA longer than 500 ms (d = −0.36). Both effect sizes were significant (see Table 1 for details). As indicated by meta-regression, the mean daily antipsychotic dosage was one of the moderators for the effect size (z = 2.10, p<0.05), suggestive of larger effect sizes in patients with higher dosages (Figure 3A).

### The amplitudes of the N400 for congruent/related conditions

Fifteen studies including 241schizophrenia patients and 241 controls were used in this part of the meta-analysis. A significant effect size of −0.55 was obtained (Z = −2.98, p<0.01), reflecting more negative N400 amplitudes for the congruent/related condition in patients with schizophrenia compared with controls. The variance was 0.004, and the 95% CI was −0.92−−0.19. These studies were not homogeneous (I-squared = 73.46%, p<0.001). The fail safe N was 105. Again, we found that the study by Guerra et al. (2009) generated an extremely large effect size (d = −3.24), which might be related to the use of pictures as experimental materials and the fact that only patients with paranoid schizophrenia were recruited.

After excluding the study by Guerra et al. (2009), the other 14 studies were found to be homogenous (I-squared = 21.77%, p = 0.22,). The effect size was −0.37, which was reliable as indicated by the test of null (Z = −3.33, p<0.001). The variance was 0.01, and the 95% CI was −0.58−−0.15. The fail safe N was 42. As a result, we excluded the study by Guerra et al. (2009) in the subsequent analysis of moderators.

As shown in Table 1, five studies with a short SOA produced an unreliable effect size of −0.17 (p>0.10). Seven studies with a long SOA produced a significant effect size of −0.52, suggestive of less efficient processing of related/congruent materials. The mean daily antipsychotic dosage could partially explain the change in effect size on meta-regression (z = −2.47, p = 0.01), reflecting larger effect sizes for patients with higher dosages (figure 3b). It is not clear whether the significant regression was mediated by symptom severity.

### Amplitudes of the N400 for incongruent/unrelated conditions

Sixteen studies including 261 patients and 261controls were used for this part of the meta-analysis, generating an effect size of −0.01. This small effect size was not reliable (Z = −0.11, p = 0.91). The variance was 0.01 and the 95% CI was −0.24−0.22. The studies in the pool were heterogeneous (I-squared = 42.64%, p = 0.04).

Five studies using a short SOA generated a small effect size of 0.24, but it was not significant (Z = 1.19, p = 0.23). It is noteworthy that these five studies showed a trend towards heterogeneity (I-squared = 53.98%, p = 0.07). Ten studies using a long SOA produced an effect size of d = −0.16, appearing to indicate more negative N400 amplitudes for incongruent/unrelated condition in the patient group. However, this effect was insignificant as indicated by the test of null (Z = −1.42, p = 0.16). All these 10 studies were homogenous (I-squared = 29.70%, p = 0.17). The mean daily antipsychotic dosage did not influence the effect sizes on meta-regression (p>0.10).

## Discussion

In this meta-analysis, we examined quantitatively four important facets of the N400 based on data extracted from carefully selected published studies to clarify the semantic processing features in patients with schizophrenia.

### Early automatic semantic activation

Studies using a short SOA generated a medium negative effect size for the N400 effect (d = −0.41) in the present meta-analysis, indicating a decreased N400 effect in patients with schizophrenia. The decrease in N400 effect suggests that patients with schizophrenia are less sensitive to the difference when activation spreads between closely related and weakly related or remotely related nodes in the semantic network. Theoretically, two possibilities could account for these findings. First, patients with schizophrenia may be impaired in processing related conditions and this may imply that either the links between related nodes are weaker or semantic activation spreads slower from one node to related nodes. Alternatively, patients with schizophrenia may have abnormality in processing weakly related or remotely related nodes such that there are unusual strong links between them. Unfortunately, the present meta-analysis could not provide a clear-cut answer. There were unreliable effect sizes of −0.16 for related condition and 0.24 for unrelated conditions and studies used for computing these two mean effect sizes were not homogenous.

In fact, the most contentious debate regarding semantic processing in patients with schizophrenia is focused on early automatic semantic activation. A relatively consistent finding is a reduction in the N400 effect [16], [25], [26], [34], [39](An exception is that Kreher et al. (2009) observed a larger N400 effect in patients with schizophrenia which was absent in healthy controls in an implicit semantic priming task.). Regarding N400 amplitudes for related conditions, some ERP studies had reported more negative amplitudes in the patient group compared with healthy controls [16], [22], [24], [34], while others found no difference [25], [26]. Similar findings have been reported for N400 amplitudes for unrelated conditions. Some studies found more negative amplitudes in patients with schizophrenia when compared to healthy controls [34], while some reported less negative amplitudes [24], [25], [26], and others found no difference [16], [22]. These diverse findings suggest that patients with schizophrenia may not have a common impaired mechanism in the early semantic activation process. The exact deficit may be modified by factors such as severity of illness, medication status, different symptoms, length of illness, and so on. For example, in a recent study, Kreher et al. (2008) observed a larger indirect N400 effect only in patients scoring higher than median of brainwave amplitudes in a group of patients with schizophrenia but not in patients scoring lower than median. Condray et al. (2003) found the N400 effect only in patients under haloperidol treatment but not in patients on placebo. As suggested by Kreher et al. (2009), the exact experimental design may also be important. They observed a N400 effect in implicit semantic tasks, but not in explicit tasks, even though the SOA in both tasks was kept constant. More research is necessary to clarify the nature of decreased N400 in the early stage of semantic processing in patients with schizophrenia.

No effect size for the N400 peak latency in studies with a short SOA could be computed, since most authors did not report this measure. Among existing studies, none reported a smaller N400 peak latency in patients with schizophrenia; one recent study reported a larger N400 peak latency in patients with schizophrenia [26]; and all of the other studies did not report any difference in the peak latency between patients and normal controls [16], [18], [22], [24], [34], [39], [40], [41]. This may suggest a relatively normal speed of automatic semantic activation in patients with schizophrenia.

### Late contextualization execution

Similarly, we obtained a negative effect size of −0.36 in the N400 effect for studies with a SOA longer than 500 ms, indicating a smaller N400 effect in the patient group. Whether the smaller N400 effect in contextualization execution was due to deficits in processing congruent context or deficits in processing incongruent context or both is unclear. Results from the meta-analyses of N400 amplitudes for congruent/related and incongruent/unrelated conditions support the first possibility. For congruent/related conditions, a reliable effect size of −0.53 was obtained for N400 amplitudes, suggesting abnormally large N400 amplitudes for contextually congruent materials; while the effect size (−0.16) for N400 amplitudes for incongruent/unrelated conditions was not significant. These findings highlighted the deficits in processing congruent context in the patient group. According to the three-process theory of Neely and Keefe, these findings indicate that patients with schizophrenia might be impaired in generating a proper lexical candidate set for a certain semantic context, and/or that they may have deficits in integrating new information with the previous semantic context. However, their inhibition mechanism to semantically incongruent materials seems to be intact.

It is interesting to note that patients with schizophrenia were not different from normal controls when they encountered words which were semantically unrelated to context (as indicated by the unreliable effect size for N400 amplitudes for incongruent/unrelated conditions). Speech in schizophrenia is characterized by ‘loosening of association’ and results from the present meta-analysis suggest that the ‘loosened association’ may be due to an inability to find proper words for expression instead of real loosened thoughts.

Results of the present meta-analysis are also consistent with findings from previous studies with homographs [27], [42], [43], idioms [44], metaphors [45] and picture word verification task [24]. Patients with schizophrenia also showed impairments in contextualization in these tasks. For example, when participants were presented with sentences like ‘The toast is sincere’, patients with schizophrenia had a larger N400 to the word ‘sincere’, indicating that they inappropriately associated the word ‘toast’ with its dominant meaning of ‘slices of brown bread’, rather than its subordinate meaning ‘to lift a glass of wine’ [27], [42], [43].

Moreover, an effect size of 0.82 for N400 peak latency was obtained for studies with a SOA longer than 500 msec. The delayed peak latency in patients with schizophrenia suggests a slower information processing speed in patients than normal controls. Lower information processing speed has been repeatedly observed in behavioral studies showing longer reaction times in patients. An interesting question is whether this slow information processing reflects a generally slow cognitive process or a specific slowness in semantic processing. To clarify this issue, many researchers compared the latency of early components, such as P1 and N2, in patients and controls, but most studies found no difference in these two components [18], [23], [30], [43], [46], [47], [48](A difference in the P2 in peak latency between patients and controls was found by Koyama et al., 1994).

### The influence of medication in semantic processing

So far seven studies have compared the correlation between N400 measures and antipsychotic dosage. Only Salisbury et al. (2000) found a correlation between antipsychotic dosage and N400 amplitudes, while the other six studies did not [16], [17], [18], [23], [26], [49]. However, the non-significant findings in these studies might be due to similar dosage in patients within the same group. Using meta-regression procedures, we found that the dosage of antipsychotic medication was a moderator for the effect sizes of N400 effect and N400 amplitudes for congruent/related conditions and there was a dose-response relationship between antipsychotic dosage and effect sizes. It is likely that the influence of antipsychotic dosage is mediated by the severity of illness.

Dopaminergic transmission might also influence semantic processing. Condray et al. (1999) recruited participants during haloperidol maintenance therapy and placebo replacement (most participants had medication history). The two groups of patients had similar severity in clinical symptoms as assessed by the Brief Psychiatric Rating Scale. A semantic priming effect in the ERP data was only found in patients on haloperidol treatment, but not in patients on placebo [39].

### Conclusions

Deficits in both early automatic semantic activation and late contextualization execution have been put forward to explain the possible deficits in semantic processing in patients with schizophrenia. Results from the present meta-analysis suggest that these views may be complementary rather than in conflict. In early automatic semantic activation, patients with schizophrenia process weakly or remotely nodes similarly to closely related nodes in their semantic network. In late contextualization execution, patients with schizophrenia appear to have problems in processing congruent context rather than incongruent context. Medication status also appears to contribute to semantic processing deficits. One limitation of this meta-analysis is that we have to group studies using different paradigm. Future studies could investigate semantic processing in the whole schizophrenia spectrum, including not only patients with established illness, but also individuals at high risk.

## Supporting Information

Table S1 **Profile of studies included in the meta-analysis of the four N400 measures.**
(DOC)
