# Supplementary material for: Semantic Processing Disturbance in Patients with Schizophrenia: A Meta-Analysis of the N400 Component
Source: PLoS One. 2011 Oct 12;6(10):e25435. doi: 10.1371/journal.pone.0025435 (PMC3192062; doi:10.1371/journal.pone.0025435)
Supplement: Table S1 — Profile of studies included in the meta-analysis of the four N400 measures. (DOC) [file pone.0025435.s001.doc]

**Table S1.** Profile of studies included in the meta-analysis of the four N400 measures

| Studies | Number of patients | Task | Medicated | Main findings in N400 measures | | | |
| --- | --- | --- | --- | --- | --- | --- | --- |
|  |  |  |  | peak latency | effect | for congruent/related | For incongruent/unrelated |
| Adams et al., 1993 ^a,b^ | 12 | Sensibility of sentence judgment | Yes | SZ > NC | Sz < Nc |  |  |
| Bobes et al. 1996 (cuba group) ^a,b^ | 20 | Semantic relatedness judgment (pictures) | Yes | SZ > NC | Sz < Nc |  |  |
| Bobes et al., 1996 (Chinese group) ^a,b^ | 20 | Semantic relatedness judgment (pictures) | Yes | SZ > NC | Sz < Nc |  |  |
| Condray et al., 1999 ^b,c,d^ (950 ms SOA, medicated group, high expectancy) | 13 | Lexical decision | Yes | SZ > NC | N.A. | N.A. | N.A. |
| Condray et al., 2003 ^b,c,d^  (350 ms SOA, medicated group, low expectancy) | 17 | Lexical decision | Yes |  | N.A. | N.A. | N.A. |
| Grillon et al., 1991 ^a,b^ | 14 | Semantic relatedness judgment (word pairs) | Yes | SZ > NC | Sz = Nc ^*^ |  |  |
| Guerra et al., 2009 ^a,b,c,d^ | 21 | Semantic relatedness judgment (pictures) | Yes | SZ > NC | Sz < Nc | Sz < Nc | Sz = Nc |
| Hokama et al., 2003 ^b^ | 18 | Lexical decision | No | SZ > NC | SZ < Nc |  |  |
| Iakimova et al., 2005 ^b,c,d^ | 20 | Sensibility of sentence judgment | Yes |  | N.A. | Sz < Nc | SZ > NC |
| Kiang et al., 2007 ^b^ | 18 | Category exemplar judgment | Yes | SZ = NC | Sz = Nc |  |  |
| Kiang et al., 2008 (SOA = 300 ms) ^b,c,d^ | 16 | Lexical decision | Yes |  | N.A. | Sz < Nc | N.A. |
| Kiang et al., 2008(SOA = 750 ms) ^b,c,d^ | 16 | Lexical decision | Yes |  | N.A. | Sz < Nc | Sz = Nc |
| Kostova et al., 2003  (Sentence based)^b,c,d^ | 12 | Lexical decision | Yes |  | N.A. | Sz < Nc | N.A. |
| Kostova et al., 2003  (word based) ^b,c,d^ | 12 | Lexical decision | Yes |  | N.A. | Sz < Nc | N.A. |
| Kostova et al., 2005 ^b^ | 50 | Lexical decision | Yes |  | SZ < Nc | Sz < Nc |  |
| Kuperbert et al., 2006 (pragmatic-semantic violations) ^a,b,c,d^ | 20 | Sensibility of sentence judgment | Yes |  | N.A. | SZ = Nc | SZ = Nc |
| Kuperberg et al., 2006  (animacy-semantic violations) ^a,b,d^ | 20 | Sensibility of sentence judgment | Yes |  | N.A. | SZ = Nc | SZ = Nc |
| Mathalon et al., 2002 ^c,d^ | 18 | Picture-word verification | Yes | SZ = NC |  | Sz = Nc | SZ < NC |
| Nestor et al., 1997 ^b,c,d^ | 18 | Sensibility of sentence judgment | Yes | SZ > NC | N.A. | Sz < Nc | Sz = Nc |
| Niznikiewicz et al., 2010 ^b,c,d^ | 19 | Lexical decision | Yes | SZ > NC | N.A. | Sz = Nc | SZ < NC |
| Ohta et al, 1999 ^a,b,cd^ | 13 | Sensibility of sentence judgment | Yes | SZ = NC | Sz < Nc | Sz < Nc |  |
| Olichney et al., 1997 (SZ with late onset) ^a,b^ | 9 | Semantic relatedness judgment ( heard phrases and visually presented words) | Yes | SZ > NC | Sz = Nc |  |  |
| Sitnikova et al., 2002 ^cd^ | 12 | Sentence-word relatedness judgment | yes |  |  | Sz = Nc |  |
| Strandburg et al.,1997 ^cd^ | 17 | Sensibility of phrase judgment | yes |  |  | Sz < Nc |  |

Note: a = used for meta-analysis of N400 peak latency; b = used for meta-analysis of N400 effect; c = used for meta-analysis of the N400 amplitudes for congruent/related conditions; d = used for meta-analysis of N400 amplitudes for incongruent/unrelated conditions.

SZ = schizophrenia; N.A. = no detailed statistic was made, but the data for meta-analysis were available; *= by peak amplitude

The blank items indicated that the data were either not given by the authors, or the given data were unable to be used in the meta-analyses.
